# Feasibility of data extraction and evaluation with BeoNet-Halle: outcomes and data quality in hepatitis B and C screenings

**DOI:** 10.3389/fmed.2025.1549032

**Published:** 2025-06-18

**Authors:** Konstantin Moser, Felix Bauch, Christine Brütting, Thomas Frese

**Affiliations:** ^1^Institute of General Practice and Family Medicine, Interdisciplinary Center for Health Sciences, Martin-Luther-University Halle-Wittenberg, Halle (Saale), Germany; ^2^Institute of Medical Epidemiology, Biometrics, and Informatics, Interdisciplinary Center for Health Sciences, Martin-Luther-University Halle-Wittenberg, Halle (Saale), Germany

**Keywords:** BeoNet-Halle, general practice, hepatitis, screening, electronic medical records, feasibility, data quality

## Abstract

**Background:**

To support the global hepatitis strategy, the Federal Joint Committee in Germany introduced a one-time hepatitis screening within the “Check-Up 35” program on 1 October 2021. Targeting individuals aged 35 and older, this preventive check-up aims to detect common diseases early. This study examines the feasibility of using the BeoNet-Halle database to characterize patients screened for hepatitis B (HBV) and hepatitis C virus (HCV), focusing on screening volumes, billing codes, and data completeness.

**Methods:**

We analyzed electronic medical records from all 11 practices contributing to the BeoNet network during the observation period from 1 October 2021 to 30 September 2023. The analysis focused on antibody and antigen tests, HBV-DNA and HCV-RNA tests, and billing codes (01734, 01744, 01865, 01866, 01867) to assess screening volumes, data completeness, and costs. Data completeness was evaluated by mapping the BeoNet dataset to the Medical Informatics Initiative (MII) Core Dataset.

**Results:**

Of the potentially eligible population (32,213 patients aged ≥ 35), 10% underwent HBV and HCV screening as part of Check-Up 35. Screened individuals had more practice contacts (mean ± SD: 22.1 ± 19.8 vs. 11.1 ± 17.7; w = 0.3) and more chronic conditions (mean ± SD: 6.9 ± 5.6 vs. 5.8 ± 6.2; w = 0.03) than the eligible practice population. Screening identified 20 new cases (0.6%), with practice-level screening rates ranging from 2.5% to 42.6%. Billing code 01734 was documented in 81.5% of cases with laboratory test billing codes (01865–01867) missing in 5 of the 11 practice management systems (PMS). The BeoNet laboratory dataset provided full coverage for test identifiers (100%) and strong documentation of result interpretations (98.7%), but had limited coverage for reference range (60.4%) and test collection dates (9.1%).

**Conclusion:**

Improving data quality and billing documentation in the BeoNet database could enhance screening accuracy and resource allocation, supporting better outcomes in hepatitis screening practices.

## Introduction

To align with the global strategy of the World Health Organization in 2016 to diagnose 90% of all HBV and HCV cases by 2030, the Federal Joint Committee in Germany implemented a one-time hepatitis screening as part of the routine Check-Up 35, effective 1 October 2021 (1, 2). The Check-Up 35 in Germany is a preventive health check-up for individuals aged 35 and older, aimed at early detection of common diseases (2). Given that only general practitioners (GPs) are authorized to perform the Check-Up 35, general practice settings play a central role in the hepatitis screening process.

The systematic use of electronic medical records from general practices for analyzing population-based screening measures remains underdeveloped in Germany. Projects like RADARplus rely on data extraction formats unsuitable for mapping screening workflows, while others, such as MedVip and CONTENT, have concluded. Consequently, hepatitis screening analysis often depends on health insurance data, which, being designed for accounting purposes, lacks critical clinical details such as information on test results, individual patient risk factors, and indications for screening (3). These health insurance databases also cannot consolidate results from multiple testing facilities or account for privately insured patients—about 13% of the German population—potentially introducing selection bias (4).

The BeoNet-Halle outpatient database, managed by the Institute of Medical Epidemiology, Biometrics and Informatics and the Institute of General Practice and Family Medicine at Martin Luther University Halle-Wittenberg, addresses these challenges. By exporting data directly from practice management system (PMS) on a monthly basis, it links laboratory results with anonymized patient data, providing a robust foundation for mapping hepatitis screening processes and enabling detailed analyses of disease detection and progression (5). Data completeness remains a critical issue, as Germany’s fragmented data infrastructure often hinders comprehensive analysis. The Medical Informatics Initiative (MII) Core Dataset serves as a reference standard for data quality, emphasizing completeness (6, 7).

The objective of this study was not to evaluate clinical effectiveness or outcomes of hepatitis screening, but rather to assess the feasibility of using the BeoNet-Halle outpatient database to characterize screening activities, identify data gaps, and evaluate the quality and completeness of electronic medical records for research purposes.

## Materials and methods

### Study design and guidelines

This study adhered to the Strengthening the Reporting of Observational Studies in Epidemiology guidelines for cohort studies. The research design is observational and retrospective, analyzing patient data to evaluate the feasibility and outcomes of HBV and HCV screenings (8).

### Database

BeoNet-Halle constitutes a network of observational health practices in primary care throughout Germany. Patient data is methodically gathered from these practices, either anonymized or pseudonymized, and then uploaded to a research database. This database is established and maintained by the Institute of Medical Epidemiology, Biometry, and Informatics, alongside the Institute of General Medicine at the Medical Faculty of Martin Luther University Halle-Wittenberg. BeoNet-Halle employs a specialized consent form, adapted from the nationally standardized broad consent frameworks of the MII (8). BeoNet Halle does not collect or use any biosamples. Patients have several consent options, such as permitting the linkage of their data across the network’s practices and healthcare facilities or agreeing to potential future re-contact.

### Study population and data extraction

All 11 general practices that were part of the BeoNet-Halle network at the time were included in the study. The *practice population* included all individuals who could potentially receive care from these practices as their primary care provider. From this group, *active patients* were identified as those with at least one recorded practice contact—defined as a billing code, prescription, or diagnosis documented in the PMS—during the observation period (1 October 2021 to 30 September 2023). This definition of a practice contact refers to any entry in the electronic medical record and does not necessarily indicate an in-person patient visit.

The study population, or potentially eligible population for hepatitis screening, comprised active patients aged 35 years or older. Prior hepatitis B or C screenings or diagnoses were not excluded, which may have influenced the proportion of patients eligible for first-time screening.

Patient data included sociodemographic details, practice contact dates, medical diagnoses, prescriptions, billing codes, lab tests, and referrals, forming the basis for evaluating hepatitis screenings.

### Definition of positive cases due to Check-Up 35

We defined a positive case due to the Check-Up 35 based on the following criteria: (1) the patient did not have a documented hepatitis diagnosis prior to HBV-DNA or HCV-RNA test within the observation period, (2) a hepatitis B or C diagnosis was recorded following a positive test result, or (3) the patient’s viral load exceeded the corresponding threshold for HBV-DNA or HCV-RNA detection. For HBV, a viral load above 2,000 IU/mL was considered clinically significant by the respective laboratories. For HCV, a detectable HCV RNA level, typically above 15–25 IU/mL depending on assay sensitivity, was used to indicate active infection according to the respective laboratories.

### Operationalization

The billing codes 01734 or 01744 for HBV and HCV screening, 01865 for the detection of HBV antigen and/or HCV antibody, and 01866 or 01867 for the determination of HBV-DNA and HCV-RNA were analyzed. These billing codes are standard for hepatitis screening. We examined the frequency of billing for these codes across all participating practices. Additionally, we analyzed the total number of screened patients per practice, the number of patient years for the screened patients, and the results of the screening tests (negative/positive).

Regarding laboratory findings, we grouped test identifiers into four main categories: antigen, antibody, and DNA or RNA test results. Due to the potential splitting of test results on the same day into multiple tests (virus load indication, interpretation of finding and so on), the table counts each test result by date, ensuring that each category (antigen, antibody, and DNA or RNA) was only counted once per day per patient.

### Data analysis

We present the patient population, stratified by sex and age, with counts and percentages. Age groups, average patient age, practice contacts, and acute and chronic conditions are presented with means and standard deviations. The calculation of patient years for the screened patients was as follows: Last billing code date in period–first billing code date in period (01.10.2021–30.09.2023). Only the absolute frequencies of the billing codes and performed screening tests were analyzed. We did not apply traditional inferential statistical measures or significance tests; instead, we utilized RD/MD, w, and η^2^ for descriptive comparisons. The statistical analyses were conducted using Python 3.10.12 in conjunction with the Pandas library for robust data manipulation and analysis.

### Assessment of missing values

To assess the data quality of laboratory findings in the BeoNet hepatitis screening dataset, we conducted a structured field mapping. The mapping aligned fields in the BeoNet dataset with the Laboratory Findings Module (version 2.0) of the MII Core Dataset (8). This module provides a standardized structure for key data elements, including Conducted Tests, Test Date, Result, Interpretation, Reference Range, and Sample Characteristics. The assessment involved calculating the percentage presence of each field in the BeoNet dataset across all records, measuring the completeness of documentation.

### Ethical approval

The study obtained ethics approval from the Martin-Luther-University Halle-Wittenberg’s researcher ethics committee (reference number: 2023-010). Ethical approval allowed the researchers to collect anonymized data from the PMS.

## Results

### Patient characteristics

From 1 October 2021 to 30 September 2023, 32,213 patients aged 35 years or older (47.1% male) had at least one contact with one of the 11 BeoNet-Halle practices, comprising the *potentially eligible population* for hepatitis screening. Among these, 3,317 patients (10.3%) were screened for HBV and HCV during the “Check-Up 35.” The screened group had a slightly higher mean age (60.1 years vs. 59.0 years) and a higher proportion of females (55.1% vs. 52.8%) compared to the total potentially eligible population (see Table 1 for details).

**TABLE 1:** Patient characteristics of potentially eligible population (PEP) (*n* = 32.213) and screened patients (S) (*n* = 3.317).

	Potentially eligible population	Screened*	PEP vs. S	
	***n* (%)/mean ± SD**	***n* (%)/mean ± SD**	**RD/MD**	**w/η^2^**
**Number**				
	32,213	3,317		
**Mean patient years per patient**				
	0.8 ± 0.8	1.5 ± 0.6	0.7 years	< 0.01
**Sex**				
Female	17,023 (52.8)	1,613 (55.1)	2.3 pp	0.03
**Age distribution**				
Mean	59.0 ± 15.0	60.1 ± 14.1	1.1 years	< 0.01
**Age groups**				
35–44	6,834 (21.2)	576 (17.4)	3.4 pp	0.08
45–54	6,371 (19.8)	610 (18.4)	1.4 pp	0.03
55–64	7,629 (23.7)	851 (25.7)	2.0 pp	0.04
65–74	5,653 (17.5)	715 (21.6)	4.1 pp	0.09
75–84	3,902 (12.1)	411 (12.4)	0.3 pp	< 0.01
85+	1,824 (5.7)	154 (4.6)	1.1 pp	0.04
**Mean practice contacts per patient (SD)**				
	11.1 ± 17.7	22.1 ± 19.8	11.0 pp	0.3
**Co-morbidities**				
Mean count of chronic conditions per patient	5.8 ± 6.2	6.9 ± 5.6	1.1 pp	0.03
Mean count of acute conditions per patient	10.4 ± 18.8	17.4 ± 18.6	7.0 pp	0.1

*Percentage of practice population screened for hepatitis B and C. Data is shown as count in numbers and frequencies (%) or as means with standard deviation. Risk difference (RD), mean difference (MD), η^2^ and Cohen’s w refer to the comparison between PEP group and S.

The age group 65–74 was overrepresented among screened individuals by 4.1 percentage points compared to the potentially eligible population. In contrast, the 35–44 age group showed an underrepresentation.

Screened individuals had more practice contacts on average (22.1 ± 19.8) than the potentially eligible population (11.1 ± 17.7), with a mean difference of 11.0 contacts (w = 0.3). The screened group also had higher mean counts of ICD-10 coded chronic and acute conditions (Table 1).

### Screening volume for hepatitis B and C screening

Table 2 provides an overview of the screening volume and billing codes used for HBV and HCV screening across the included practices. The overall corrected screening rate, defined as the percentage of patients screened for both viruses, was 10.0% of the potentially eligible population (*n* = 32,213). Targeted screening, where only one of the HBV or HCV antibodies was tested, occurred in 0.3% of those screened, while multiple screenings, where both viruses were screened more than once, occurred in 0.3% of the potentially eligible population.

**TABLE 2:** Hepatitis B and C screening and billing codes in 11 general practices (01.10.2021–31.09.2023).

Practice	Population (> 35 years)	% Corrected screening rate^1^	% Multiple screenings^2^	% Exclusively screened for Hep B or C^3^	% With billing code^4^	No. with DNA determination for Hep B^5^ (viral load indication)	No. with RNA determination for Hep C^6^ (viral load indication)
1	450	9.1%	0.7%	0.0%	51.2%	0	0
2	3,853	11.3%	0.3%	0.2%	87.8%	2 (1)	5 (2)
3	4,334	14.4%	0.8%	0.7%	77.2%	5 (3)	2 (1)
4	999	42.6%	0.0%	0.1%	85.7%	2 (1)	3 (1)
5	2,715	9.1%	0.5%	0.2%	56.5%	1 (1)	1 (0)
6	1,790	7.4%	0.2%	0.2%	93.9%	1 (1)	0
7	1,705	30.0%	0.8%	0.2%	85.4%	2 (1)	0
8	1,398	5.4%	0.2%	0.1%	68.0%	0	0
9	7,984	2.5%	0.1%	0.1%	80.0%	2 (1)	0
10	3,045	17.3%	0.0%	0.3%	90.3%	1 (1)	3 (2)
11	3,940	0.4%	0.0%	0.2%	0.0%	1 (0)	0
Total	32,213	10.0%	0.3%	0.3%	81.5%	17 (10 + 2^7^)	14 (6 + 2^8^)

^1^Percentage of practice population screened for both hepatitis B and C as part of the check-up.

^2^Percentage of practice population screened more than once (> 1) for both hepatitis B and C antibodies as part of the check-up.

^3^Percentage of screened patients where either a hepatitis B or C antibody test was done.

^4^Percentage of screened patients who received a hepatitis B and C check-up billing code (01734 or 01744).

^5^Number of cases with hepatitis B DNA determination and virus load indication.

^6^Number of cases with hepatitis C RNA determination and virus load indication.

^7^Among 12 hepatitis B cases identified through Check-Up 35, 10 had viral load reported, and 2 were qualitatively positive for HBV DNA.

^8^Among 8 hepatitis C cases identified through Check-Up 35, 6 had viral load reported, and 2 were qualitatively positive for HCV RNA.

Among the 3,317 screened patients, HBV-DNA determination was performed for 17 patients, and HCV-RNA determination for 14 patients. Quantitative virus load measurements were available in 10 cases for HBV-DNA and in six cases for HCV-RNA. A qualitative result for the viral load was reported for four cases (2 for HBV 2 for HCV). So in total, 20 new cases of HBV or HCV were identified through the Check-Up 35, corresponding to 0.60% of the correctly screened population.

Screening rates varied considerably among practices, ranging from 2.5% to 42.6%. One practice, with a screening rate of 0.4%, was excluded as it only provided data for the first six months of the observation period. All other practices contributed data for the full duration of the study.

### Billing code utilization and actual vs. potential billing

Billing data (Table 2) showed that the appropriate billing codes for HBV and HCV screenings, along with the check-ups (01734 or 01744), were documented for 81.5% of screened patients. This translated into actual costs of €12,838.40. However, the full potential billing amount was calculated at €15,752.60, resulting in €2,914.20 in forgone revenue. On average, each practice experienced a revenue loss of €264.20 due to under-billing.

In 5 out of 11 practices, the necessary billing codes for critical HBV and HCV tests, such as HBs antigen and/or HCV antibody detection (01865), hepatitis B virus DNA (01866), and hepatitis C virus RNA (01867), were not provided.

### Field coverage of MII laboratory findings in BeoNet hepatitis screening data

The BeoNet dataset from 11 general practices demonstrated full coverage for laboratory test identifiers (100%) but limited documentation for test status (9.2%) and lab test collection date (9.1%) (Table 3). Result fields showed mixed coverage, with Result Text and/or Interpretation well-documented (98.7%), but result value rarely recorded (1.2%), possibly due to the absence of values for negative results. Reference Text, which may include a reference range, was present in 60.7% of cases, while lower and upper limits were scarcely recorded (0.2% and 0.4%, respectively). Scale Type documentation was sparse (11.4%). Sample characteristics showed moderate coverage for material type (70.1%), and Source Laboratory data, an optional field in the MII module, was present. Overall, while identifiers and interpretations were strongly documented, significant gaps were identified in reference ranges, test timing, and sample data.

**TABLE 3:** Comparison of MII Core Dataset laboratory findings module with BeoNet dataset: hepatitis screening data from 11 practices.

MII Core Dataset	BeoNet dataset (Avg. % presence)
Conducted test	Test Type^1^ Test identifier^2^ (100%), test designation^3^ (100%), test status^4^ (9.2%)
Test date	Collection date^5^ (9.1%)
Results and interpretation	Result value (1.2%), result and/or interpretation text^6^ (98.7%)
Reference range	Reference value^7^ (1.2%), reference text^8^ (60.7%), reference lower limit (0.2%), reference upper limit (0.4%)
Sample characteristics	Sample material ID^9^ (70.1%), sample material description^10^ (33.8%)
Scale type	Measurement unit (11.4%)
Source laboratory	Laboratory name (100%)

^1^The report stage, such as final report, partial report or preliminary report.

^2^The unique identifier for a specific laboratory test based on a standardized coding system like LOINC.

^3^The name or designation of the test that was conducted.

^4^The report status, such as final report, already reported, corrected value or missing/to follow.

^5^The date (YYYY-MM-DD) on which the sample was collected from the patient.

^6^A qualitative or coded interpretation of the result, indicating whether it is within normal ranges or pathological.

^7^A general reference measurement used to compare against normal or expected values, indicating levels such as slightly elevated, significantly decreased, or abnormal.

^8^Text providing additional reference information, often detailing normal or acceptable ranges.

^9^A unique identifier for the sample material (e.g., blood, urine).

^10^A description of the type of sample material collected.

## Discussion

### Summary of the main findings

Correct screening for both HBV and HCV as part of the Check-Up 35 was performed in 10% of the total practice population resulting in 13 new HBV and 7 new HCV diagnoses. Billing codes for performed lab tests (01865-67) were missing in 5 of 11 practices. The BeoNet dataset demonstrated complete coverage for test identifiers (100%) and strong documentation for result interpretations (98.7%), reference range text (60.4%) but not for test collection date (9.1%).

### Discussion of the main findings

The relatively low number of HBV and HCV screenings may partly stem from structural limitations: The Check-Up 35 is a one-time screening opportunity for individuals aged 35 and older and is exclusively available through GPs (2). This setup could limit access for patients who either do not maintain regular contact with GPs or are primarily seen by specialists. Regional disparities in GP availability may further exacerbate unequal access (4). While it is possible that some patients had already been tested previously, current evidence suggests that public awareness of hepatitis screening remains limited in Germany (9, 10), indicating that low screening rates are not solely due to prior testing. In addition, existing evidence indicates that hepatitis screening is still relatively unknown in Germany (9, 10). Compared with data from other countries where, for example, only 2.8% of patients in England were screened for hepatitis B by GPs, the testing rate in Germany can be rated as quite high (11). In addition, in many countries, only risk groups are tested, which makes a comparison with German data difficult (12). The slightly higher average age of the screened patients compared to the general practice population aligns with existing evidence: Hepatitis screening was conducted for the first time, particularly in older age groups, to counter the underreporting of previously undetected HBV or HCV cases in older people (13, 14). Screened patients had more frequent interactions with the general practice which is also in line with other studies (15). Since older people and women go to the doctor more often than younger ones, this could explain the slightly higher age and higher proportion of females in the screened group (16, 17).

Notably, very few new infections were detected among patients correctly screened for HBV and HCV at the Check-Up 35. These findings are only partially consistent with existing literature. A screening for HBsAg, anti-HCV, and HCV RNA in over 21,000 patients showed a prevalence of 0.52, 0.95, and 0.43%, respectively and in 85% of HBsAg-positive and 65% of anti-HCV-positive people, the infections were previously unknown (18).

Recent studies from Germany found that since the introduction of hepatitis screening as part of the Check-Up 35 in 2021, significantly more positive HBV and HCV cases were detected compared to 2020. This trend continued in 2022 and 2023, with more positive hepatitis tests detected than in the previous year. The discrepancy in the results could at least partially be explained by the origin of the data. Previous studies were based on billing data from health insurance companies, which can show the total number of positive tests but cannot differentiate between new, previously unknown infections and already existing and known positive hepatitis cases. This is understandable, as the data was primarily collected for billing purposes. Although this makes it possible to achieve high case numbers, the informative value of routine data analyses may be limited (19, 20).

However, with the BeoNet data, which allows for the tracking of individual patients and diagnostic histories, such a differentiation is possible. Analysis of the BeoNet data at the practice level showed that individual practices varied widely in their screening for HBV and HCV, and also in their billing behavior. This could be an indication that doctors attach less importance to hepatitis screening or that the patient clientele differs significantly between the individual practices, for example with regard to the frequency of risk groups (21). At the same time, this could also be related to the above mentioned situation where patients are only screened once, which may not necessarily have taken place during the examination period. In addition, this could indicate an irregular and regionally and structurally varying screening process within German general practice settings, which could hinder the widespread identification of previously undiagnosed hepatitis cases (9).

Another finding of the study was that although the majority of HBV and HCV screenings were billed as a service, some practices did not document them correctly. Missing or incorrectly mapped GP treatment data must be taken into account in the data export prior to secondary research (22). The data structure of the BeoNet database makes it possible to check and evaluate the documentation and billing behavior of individual practices. For practices participating in BeoNet, this provides the opportunity to review their own documentation behavior and to make adjustments if necessary. This also applies, for example, to the requirements for individual laboratory parameters, which can be reconstructed from the laboratory and billing data in BeoNet.

### Strengths and limitations

This study addresses an important topic with high relevance for further research in real-world hepatitis screening. To date, there is little evidence on the effectiveness and costs of screening for HBV and HCV at the level of individual GP practices. The use of BeoNet data makes it possible to analyze screening at the level of individual patients using the findings in order to clarify cases. This makes it possible to describe which positive hepatitis cases were actually newly detected. In addition, BeoNet data makes it possible to examine information on individual practices and to classify the billing and documentation behavior for HBV and HCV of the practices.

However, there are limitations to this study. This is a small, regional sample, primarily from central Germany, which limits the generalizability of the findings, particularly to other countries. One weakness is that there is a certain lack of clarity when individual tests have been carried out, as it is not possible to determine beyond doubt why certain tests were carried out. The documentation of the parameters in the individual practices and the associated data quality may have influenced or distorted the results. By not excluding people who had already been tested for or diagnosed with hepatitis B and C, this may have affected the proportion of patients who were eligible for initial screening. Although we identified 32,213 patients aged 35 and older as the potentially eligible population, we were unable to determine how many of these individuals had previously undergone hepatitis B or C screening, which limits our ability to assess the true number of patients eligible for first-time testing. One limitation of our dataset is the lack of documentation of the specimen collection date in approximately 90% of cases. While this restricts our ability to reconstruct the exact testing process and sequence, the date of result reporting can serve as a reasonable proxy for the timing of hepatitis screenings in the context of our research question. Therefore, we do not consider this a major limitation for the present analysis, although it may be relevant for studies with a stronger focus on care pathways or process evaluation. Another limitation is the poor availability of viral load data which reduces the clinical interpretability of negative and positive cases likewise and constrains inferences about disease severity and treatment needs. Furthermore, the dataset includes only limited demographic information, such as age and sex, and lacks key socioeconomic variables such as race or ethnicity, income, family status, and educational attainment, which are not routinely documented in PMS. Therefore, the dataset lacks key clinical variables such as individual risk factors for hepatitis infection (e.g., migration background, substance use history) and hepatitis B vaccination status, which limits the ability to assess the appropriateness of screening and interpret results in a clinical context.

## Implications for practice and further research

The results of this study indicate that screening for HBV and HCV differs between GP practices and that the number of patients screened varies widely. Further research could shed light on the parameters that determine how many patients are screened. In addition, it could be shown that the service codes required for billing hepatitis tests were sometimes not documented correctly. Here, the BeoNet data can help participating practices to better understand and, if necessary, improve their documentation.

To improve data quality, standardized documentation procedures and automated prompts in practice management systems should be implemented. Feedback reports and benchmarking may help raise awareness. Incentives such as Continuing Medical Education credits or participation-based funding could motivate practices to improve data capture. Follow-up studies are planned to explore barriers to screening, assess patient follow-up, and expand the BeoNet-Halle network.

## Data Availability

The raw data supporting the conclusions of this article will be made available by the authors, without undue reservation.
